# Assessing the potential impact of salidroside on Chikungunya virus-induced acute interstitial nephritis via network pharmacology, molecular docking and *in vitro* experiments

**DOI:** 10.3389/fcimb.2025.1623860

**Published:** 2025-07-22

**Authors:** Sheng Cheng, Jialiang Xin, Tianran Zhang, Yulin Zhang, Chengxi Ji, Lulu Kang, Xiangyu Zhu, He Zhang, Wei Wang, Xinfei Liao

**Affiliations:** ^1^ Key Laboratory of Animal Disease and Human Health of Sichuan Province, College of Veterinary Medicine, Sichuan Agricultural University, Chengdu, China; ^2^ The Humanities Laboratory for Ecological Civilization and Environmental Governance of Zhejiang Province, Institute of Virology, Wenzhou University, Wenzhou, China; ^3^ Changchun Veterinary Research Institute, Chinese Academy of Agricultural Sciences, Changchun, China; ^4^ Wenzhou Municipal Center for Disease Control and Prevention, Wenzhou Municipal Institute of Health Supervision, Wenzhou, China; ^5^ Wenzhou Polytechnic, Wenzhou, China

**Keywords:** CHIKV, salidroside, network pharmacology, molecular docking, ferroptosis, apoptosis

## Abstract

Chikungunya virus (CHIKV) infection is often linked to acute interstitial nephritis (AIN) in fatal cases. Given the global spread of CHIKV and the lack of targeted antiviral treatments, there is an urgent need for effective therapeutic strategies against CHIKV-induced AIN. This study explored the therapeutic potential of salidroside (Sal) using an integrative approach involving network pharmacology, molecular docking and *in vitro* validation. Network pharmacology analysis identified 18 overlapping targets between Sal and AIN, including TNF, IL6 and AKT1. Molecular docking revealed strong binding affinities between Sal and key pathway proteins (Vina scores < –6), notably TNF, IL6 and BCL2. *In vitro* assays using CHIKV-infected 293T cells demonstrated that Sal (7.8125–2000 μM) enhanced cell viability by 8.9–25.9%, with the greatest effect observed at 1000 μM, without significantly altering viral replication. Mechanism analysis using the KEGG and FerrDB databases implicated apoptosis and ferroptosis in CHIKV-induced AIN pathogenesis. RT-qPCR analysis confirmed that Sal significantly downregulated ferroptosis-related genes (IL-6, IL-1β, SIRT1, PARP1, HMOX1) and apoptosis-associated markers (Bax, TNF-α, PARP1) in infected cells. Consistent with these findings, molecular docking demonstrated that Sal binds strongly to the ferroptosis-related protein GPX4 (Vina score: –6.3) and the apoptosis regulator NFKB1 (Vina score: –6.0). These results suggest that Sal is a promising therapeutic candidate for the treatment of CHIKV-induced AIN.

## 1 Introduction

Chikungunya virus (CHIKV) is a mosquito-borne arbovirus belonging to the genus Alphavirus (family Togaviridae) (Soumahoro et al., 2009). It is primarily transmitted to humans *via* infected Aedes aegypti and Aedes albopictus mosquitoes (Rougeron et al., 2015). The viral genome is a ~12 kb positive-sense single-stranded RNA that encodes four non-structural proteins (nsP1–nsP4) involved in replication, and three structural proteins (C, E1 and E2) essential for viral entry and assembly (Silva and Dermody, 2017).

CHIKV is endemic in more than half of the world’s regions, with over four million infections reported annually (Staples et al., 2009; Rougeron et al., 2015). Clinically, infection manifests as an acute febrile illness characterised by high fever, severe polyarthralgia, myalgia, headache and rash (Vairo et al., 2019). Although most patients recover within one to two weeks, approximately 30–40% develop chronic arthralgia that can persist for months or even years, contributing to significant morbidity and economic burden in endemic areas (Vu and Jungkind, 2017; Martins et al., 2020). The pathogenesis of CHIKV involves complex virus-host interactions. The virus infects dendritic cells, macrophages, fibroblasts and endothelial cells, eliciting a pronounced inflammatory response characterised by elevated levels of TNF-α, IL-6 and IL-1β (Mathew et al., 2017). Recent studies suggest that CHIKV can cause systemic complications, including renal dysfunction. In fatal cases, acute interstitial nephritis (AIN) is frequently observed and is believed to result from direct viral infection of renal cells, cytokine storm-induced injury and oxidative stress-mediated apoptosis (Joubert et al., 2012; Mercado et al., 2018; Aurore et al., 2021; Morel et al., 2024). However, no targeted therapies are currently available for treating AIN in CHIKV-infected individuals.

Salidroside (Sal), a phenylethanoid glycoside derived from *Rhodiola rosea*, possesses anti-inflammatory, antioxidant and immunomodulatory properties. It reduces oxidative stress by activating the Nrf2/HO-1 pathway and suppresses inflammation via inhibition of NF-κB and MAPK signalling pathways (Wang et al., 2019; Zhang et al., 2021). Moreover, Sal has demonstrated renoprotective effects in various disease models (Liu et al., 2024).

This study aims to evaluate the therapeutic potential of Sal against CHIKV-induced AIN using an integrative strategy. First, network pharmacology will be employed to identify key molecular targets and pathways implicated in CHIKV-associated AIN. Next, molecular docking will assess the binding interactions between Sal and critical host proteins. Finally, *in vitro* experiments using CHIKV-infected 293T cells will be conducted to investigate the cytoprotective effects of Sal and elucidate its underlying mechanisms.

## 2 Materials and methods

### 2.1 Cell culture, viral strain and compounds

293T and BHK-21 cell lines (China Center for Type Culture Collection, CCTCC) were cultured in Dulbecco’s Modified Eagle Medium (DMEM; Gibco) supplemented with 10% fetal bovine serum (FBS; Gibco), 100 U/mL penicillin and 100 μg/mL streptomycin (Thermo Fisher Scientific). Cells were maintained at 37 °C in a humidified incubator with 5% CO_2_.

The CHIKV strain (GenBank: MT933041.1; provided by the Changchun Veterinary Research Institute) was stored at −80°C. Sal (MedChemExpress; CAS No. 10338-51-9; purity ≥99.9%) was dissolved in sterile distilled H_2_O to prepare a 100 mM stock solution.

### 2.2 Network pharmacology-based analysis

#### 2.2.1 Targets acquisition

A total of 706 CHIKV-related targets were obtained from our previous study (Xin et al., 2025). Targets associated with acute interstitial nephritis (AIN) were retrieved from DisGeNET (https://disgenet.com), GeneCards (https://www.genecards.org) and OMIM (https://www.omim.org).

Sal-associated targets were identified by querying ‘Salidroside’ and ‘10338-51-9’ in TCMSPS (https://old.tcmsp-e.com/tcmsp.php), CTD (https://ctdbase.org), Swiss Target Prediction (http://www.swisstargetprediction.ch/), Herb (https://herb.ac.cn), with results filtered for Homo sapiens. All identified targets were consolidated and duplicate entries were removed.

#### 2.2.2 Disease and disease-compound intersection target analysis

Overlapping targets between CHIKV-induced AIN and Sal were identified. Protein–protein interaction (PPI) networks were constructed using the STRING database (Version 12.0; confidence score ≥0.9; species: *Homo sapiens*) and visualised in Cytoscape (Version 3.9.1). Core targets were ranked using four topological algorithms implemented via the CytoNCA plugin in Cytoscape. Gene Ontology (GO) and Kyoto Encyclopedia of Genes and Genomes (KEGG) pathway enrichment analyses were conducted on the intersection targets.

#### 2.2.3 Molecular docking validation

The three-dimensional structures of target proteins were retrieved exclusively from the PDB (https://www.rcsb.org), focusing on *Homo sapiens* with (< 3 A) X-ray crystallography structures.

The two-dimensional structure of Sal was downloaded from PubChem (https://pubchem.ncbi.nlm.nih.gov/). Structure-based blind docking was performed using CB-DOCK2 (http://cadd.labshare.cn/cb-dock2/php/index.php). Docking results were evaluated based on binding affinity, with particular emphasis on complexes exhibiting the lowest Vina scores.

### 2.3 Experimental validation

#### 2.3.1 Cytotoxicity assay

The 50% cytotoxic concentration (CC_50_) of Sal was determined in 293T cells seeded in 96-well plates. Cells were treated with varying Sal concentrations (15.625 to 4000 μM) in DMEM supplemented with 2% FBS for 48 h. Cell viability was assessed using the CCK-8 assay (Vazyme, A311-01, China).

#### 2.3.2 Antiviral activity

293T cells were infected with CHIKV (MOI 0.01) in the presence of different Sal concentrations. After 24 h of incubation, cell viability was measured using the CCK-8 assay. The percentage of viability was calculated using the formula: [(As-Ab)/(Ac-Ab)] × 100, where As, Ab and Ac represent the sample, blank and control absorbances, respectively.

#### 2.3.3 Viral replication

CHIKV-infected 293T cells (MOI 0.01) were treated with Sal (250–1000 μM) or left untreated as controls. After 24 h, viral RNA levels in the culture supernatants were quantified by RT-qPCR. Viral infectivity was further assessed by plaque assay on BHK-21 cells. Primer sequences are listed in **Supplementary Table 1**.

#### 2.3.4 Proliferation assay

293T cells at 30% confluence were treated with Sal at concentrations ranging from 7.8125 to 2000 μM, or left untreated as controls and incubated for 48 h. Cell viability was subsequently assessed using the CCK-8 assay.

### 2.4 Analysis of ferroptosis and apoptosis

To investigate the involvement of apoptosis in CHIKV-induced AIN, apoptosis-related genes enriched in the KEGG pathway were randomly selected and analysed using RT-qPCR across treatment groups.

Ferroptosis targets from FerrDB (http://www.zhounan.org/ferrdb/current/) and compared with CHIKV-AIN and Sal-associated targets using Venn analysis. Core ferroptosis-related targets were identified using Cytoscape (v3.9.1). Molecular docking and RT-qPCR were employed to validate the predicted targets. Primer sequences are provided in **Supplementary Table 1**.

### 2.5 Statistical analysis

Statistical analyses were conducted using GraphPad Prism (v9.5). Unpaired t-tests were used for comparisons between two groups, and one-way ANOVA was applied for multiple group comparisons. A *p*-value < 0.05 was considered statistically significant.

## 3 Results

### 3.1 CHIKV-induced AIN involves multiple pathogenic pathways

**Figure 1:**
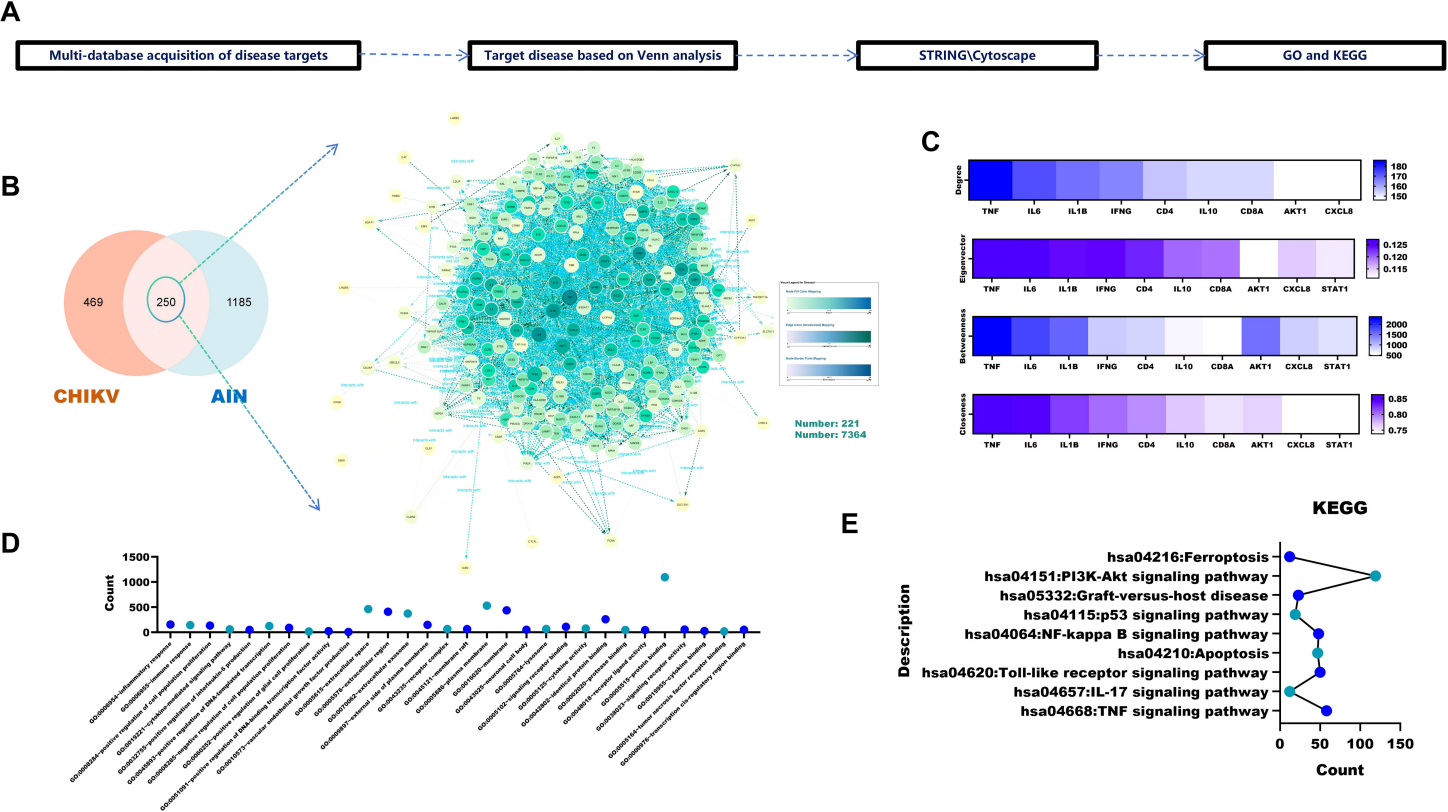
Pathogenic pathways and targets of CHIKV-induced AIN. **(A)** Acquisition and analysis of CHIKV-induced AIN targets. **(B)** Identification of CHIKV-induced targets *via* Venn analysis. **(C)** Analysis of core disease targets using four algorithms in Cytoscape 3.9.1 **(D)** GO analysis of core targets. **(E)** KEGG analysis of the core targets.

The workflow for identifying targets associated with CHIKV-induced acute interstitial nephritis (AIN) is shown in **Figure 1A**. Venn analysis revealed 250 overlapping targets between 719 CHIKV-related and 1,435 AIN-related targets (**Figure 1B**). Core targets were ranked using four topological algorithms (Betweenness, Closeness, Degree and Eigenvector) within Cytoscape 3.9.1 and the CytoNCA plugin, identifying TNF, IL6 and IL1B as the top three nodes (**Figure 1C**). GO enrichment analysis indicated that these targets are primarily involved in inflammatory and immune responses, as well as the positive regulation of cell proliferation (**Figure 1D**). KEGG pathway analysis identified ferroptosis, the PI3K-Akt signalling pathway and apoptosis as key pathways implicated in CHIKV-induced AIN (**Figure 1E**).

### 3.2 Sal has potential anti-AIN effects against CHIKV

**Figure 2:**
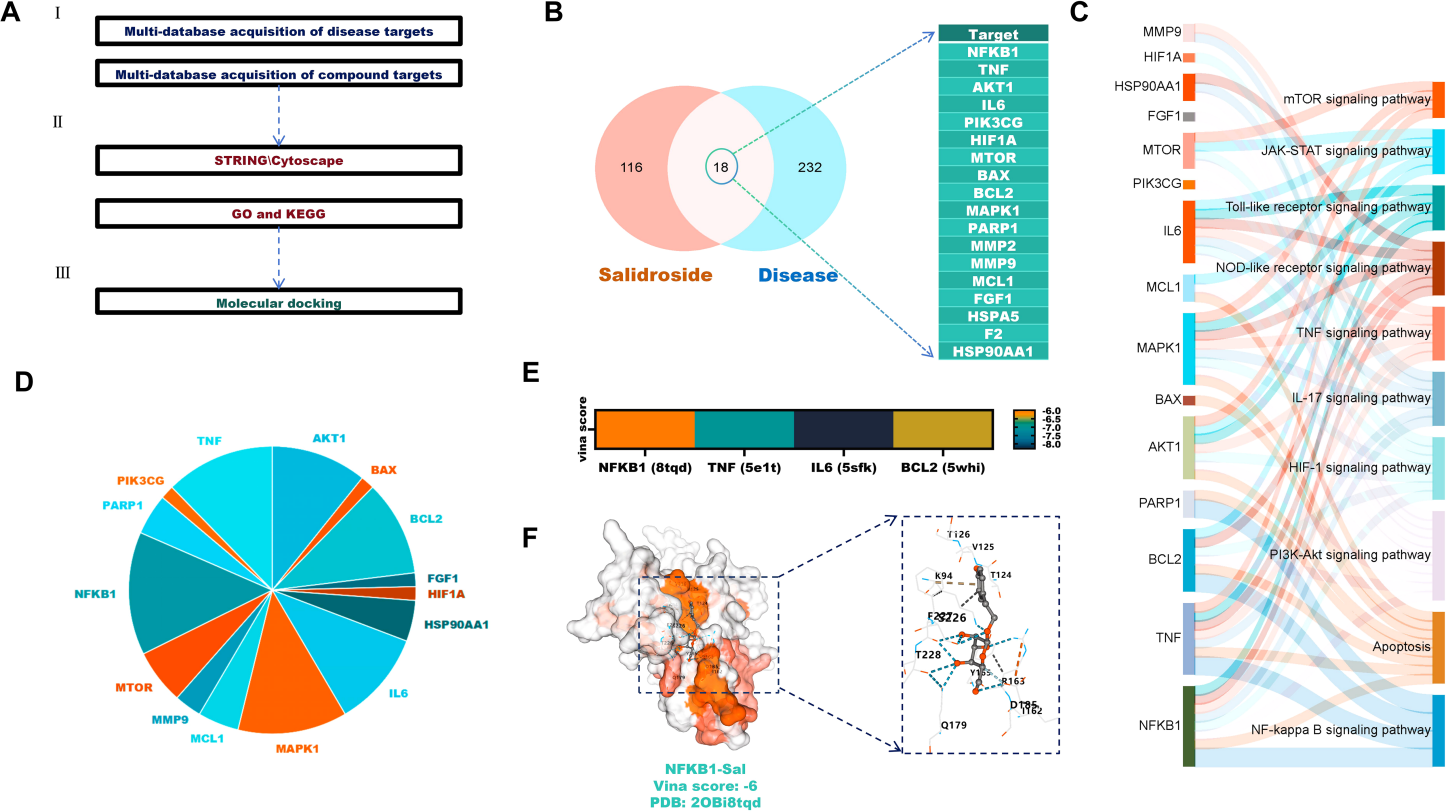
Anti-AIN potential of Sal against CHIKV. **(A)** Analysis of Sal’s disease-targeting effects **(B)** Venn analysis identifies overlapping targets between Sal and the disease. **(C)** KEGG analysis of overlapping targets. **(D)** Frequency analysis of the overlapping targets in KEGG. **(E)** Molecular docking verification. **(F)** Visualisation of the molecular docking between Sal and NFKB1 proteins.

To evaluate the potential role of Sal in treating CHIKV-induced AIN, network pharmacology analysis was conducted (**Figure 2A**). Venn analysis of 124 Sal-associated targets and 250 disease targets identified 18 overlapping targets, including TNF, IL6 and AKT1 (**Figure 2B**).

KEGG enrichment analysis showed that these targets are primarily involved in the PI3K-Akt, apoptosis and NF-κB signalling pathways (**Figure 2C**). Frequency analysis highlighted NFKB1, TNF, IL6 and BCL2 as the most frequently involved targets (**Figure 2D**). Molecular docking confirmed strong binding affinities between Sal and these proteins, with Vina scores below -6 (**Figures 2E, F**).

### 3.3 Sal enhances the viability of CHIKV-infected 293T cells

**Figure 3:**
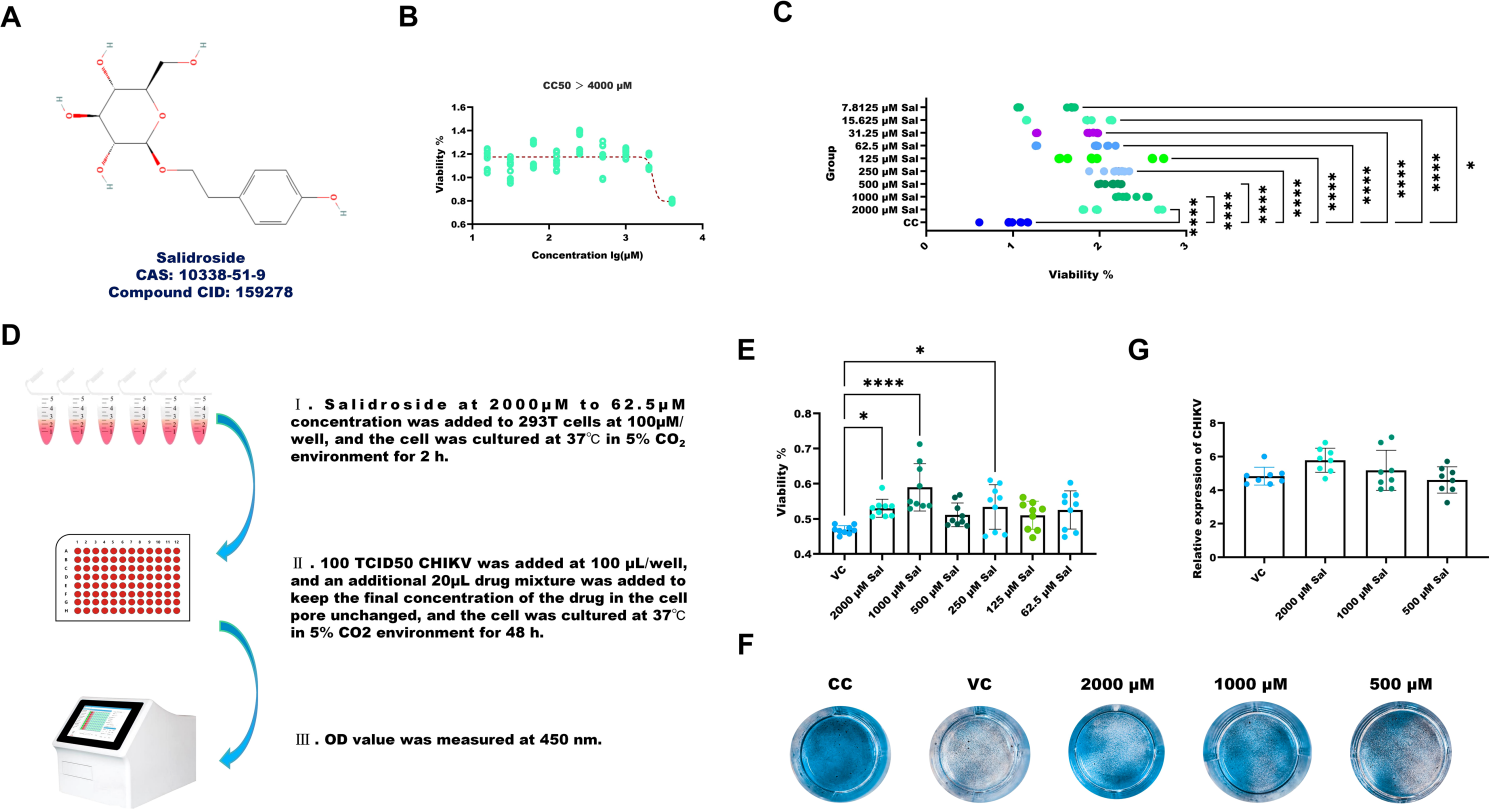
Sal enhances the viability of CHIKV-infected 293T cells. **(A)** Chemical structure of Sal. **(B)** Determination of CC_50_. **(C)** The results of Sal promoting the viability of 293T cells. **(D)** Experimental design for assessing the protective effects of Sal in CHIKV-infected 293T cells. **(E)** The results of Sal inhibiting CHIKV-induced cytotoxicity in 293T cells. **(F)** Crystal violet staining results. **(G)** Determination of CHIKV copy number in the supernatant of CHIKV-infected 293T cells treated with 1000 to 250 μM Sal. VC, Virus infection control; CC, Cell control. All values represent the mean ± SD. Compared with VC or CC, ****p < 0.0001, ***p < 0.001, **p < 0.01, *p < 0.05.

The chemical structure of Sal is presented in **Figure 3A**. Cytotoxicity assays indicated that Sal was non-toxic to 293T cells at concentrations up to 2,000 μM (**Figure 3B**). Moreover, Sal (7.8125 μM–2,000 μM) promoted cell proliferation in a dose-dependent manner (**Figure 3C**).

The experimental setup for assessing the protective effects of Sal in CHIKV-infected 293T cells is illustrated in **Figure 3D**. Treatment with Sal (250–2,000 μM) significantly improved the viability of infected cells, as measured by CCK-8 assay (**Figure 3E**) and further supported by crystal violet staining (**Figure 3F**). However, RT-qPCR analysis showed that Sal did not reduce CHIKV RNA levels, indicating that its protective effects are not due to inhibition of viral replication (**Figure 3G**).

### 3.4 The anti-CHIKV-induced AIN effect of Sal is associated with ferroptosis and apoptosis

**Figure 4:**
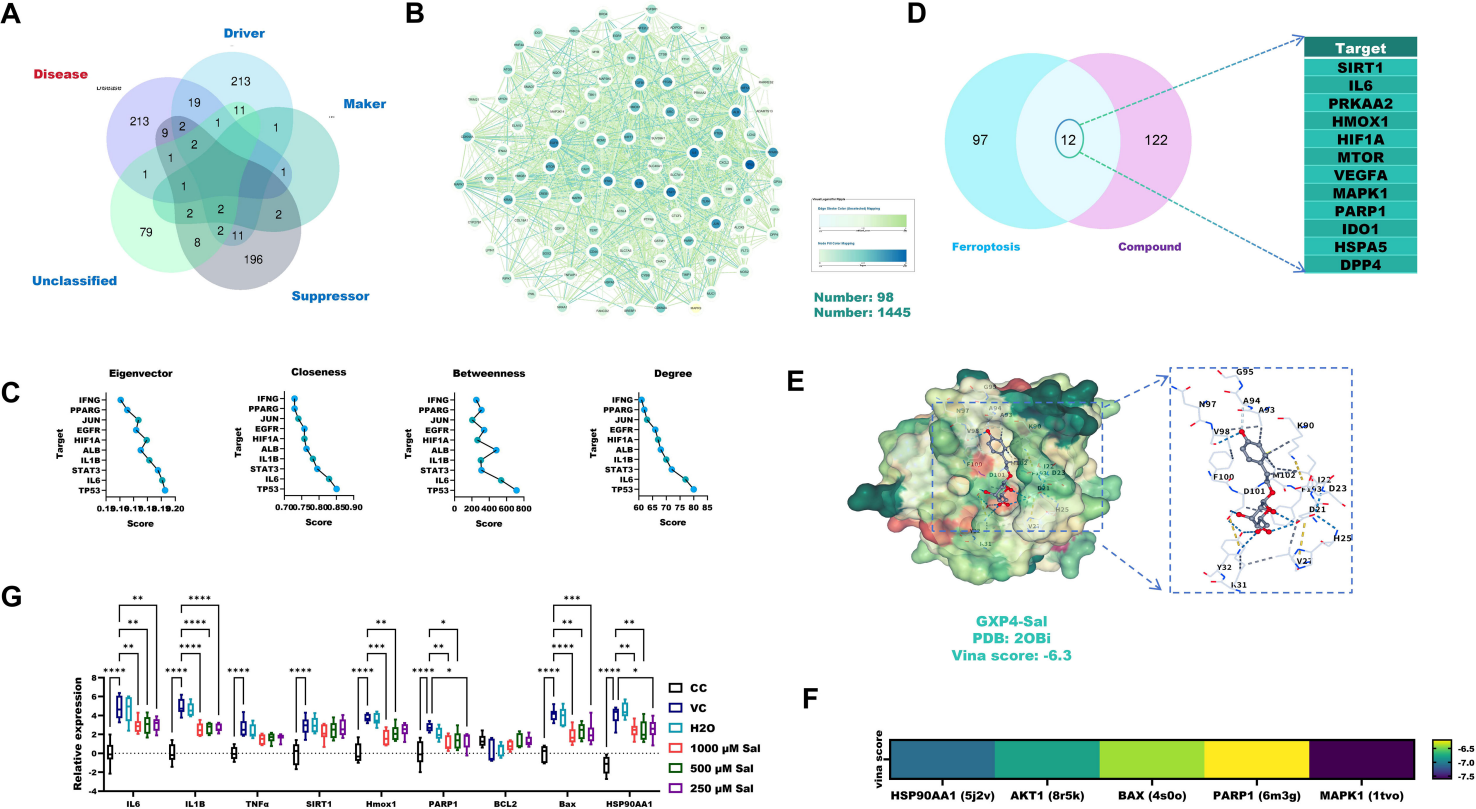
The anti-CHIKV-induced AIN effect of Sal is associated with ferroptosis and apoptosis. **(A)** Venn analysis identified overlapping targets between CHIKV-induced AIN and ferroptosis-related targets. **(B)** PPI analysis of overlapping targets. **(C)** Core target analysis of overlapping targets using Cytoscape 3.9.1 and CytoNCA. **(D)** Predicted targets of Sal against ferroptosis-related targets of CHIKV-induced AIN. **(E)** Molecular docking details between Sal and GPX4 proteins. **(F)** Molecular docking verification. **(G)** RT-qPCR verification. VC, Virus infection control; CC, Cell control. All values represent the mean ± SD. ****p < 0.0001, ***p < 0.001, **p < 0.01, *p < 0.05.

Venn analysis identified 109 overlapping targets between CHIKV-induced AIN and ferroptosis-related genes (**Figure 4A**). Core target analysis using Cytoscape 3.9.1 and the CytoNCA plugin identified TP53, IL6 and STAT3 as the top three key regulators (**Figures 4B, C**). Notably, 12 out of 18 (66.7%) predicted Sal targets against CHIKV-induced AIN overlapped with ferroptosis-associated genes (**Figure 4D**).

Molecular docking analysis revealed a strong binding affinity between Sal and GPX4 (Vina score: –6.3) (**Figure 4E**). Sal also exhibited high binding affinities (Vina scores < –6.5) with additional key targets, including HSP90AA1, AKT1, BAX, PARP1 and MAPK1 (**Figure 4F**). RT-qPCR analysis confirmed that Sal (250–1000 μM) significantly downregulated the expression of ferroptosis-related genes (IL-6, IL-1β, SIRT1, PARP1, HMOX1), apoptosis-associated markers (BAX, TNF-α, PARP1) and HSP90AA1 in CHIKV-infected 293T cells (**Figure 4G**).

## 4 Discussion

Renal impairment is a common and clinically significant complication in severe cases of Chikungunya virus (CHIKV) infection, with acute interstitial nephritis (AIN) identified as the predominant pathological manifestation (Mercado et al., 2018). Although the precise mechanisms underlying CHIKV-induced AIN remain incompletely understood, existing evidence implicates an excessive inflammatory response, particularly the upregulation of pro-inflammatory cytokines such as IL-1β, IL-6 and TNF-α during the acute phase of infection (Ng et al., 2009). Our findings support this hypothesis. Through network topological analysis, IL1B, IL6 and TNF emerged as central molecular targets associated with CHIKV-induced AIN. This prediction was validated in our *in vitro* model, where CHIKV-infected 293T cells exhibited significant transcriptional upregulation of IL-1β, IL-6 and TNF-α. Bioinformatic analysis further revealed that these cytokines are functionally interconnected with key signalling pathways, including NF-κB, PI3K-Akt and ferroptosis, suggesting a synergistic role in promoting renal injury.

Given the well-documented anti-inflammatory and renoprotective properties of Sal (Pu et al., 2020), we hypothesised that it might exert therapeutic effects against CHIKV-induced AIN. This was evaluated using our established 293T CHIKV infection model. Sal treatment (7.8–2000 μM) significantly improved cell viability in infected cells, with the most pronounced protective effect observed at 1,000 μM. Integrating network pharmacology and experimental data, we identified HSP90AA1 as a key target of Sal. RT-qPCR analysis confirmed that Sal (1000 μM) significantly downregulated HSP90AA1 expression in CHIKV-infected cells. As HSP90AA1 is known to stabilise the CHIKV non-structural protein NSP2 and facilitate viral replication (Nam et al., 2021), its inhibition was expected to reduce viral load. However, contrary to this expectation, quantitative RT-qPCR showed no significant difference in viral RNA levels between Sal-treated and untreated cells. This apparent paradox suggests that while Sal may disrupt HSP90AA1-mediated support of viral replication, compensatory mechanisms might maintain viral propagation. These could involve alternative host pathways that offset the antiviral impact of HSP90AA1 inhibition.

Several mechanisms may explain this unexpected finding. First, our network pharmacology analysis indicated that Sal is involved in the PI3K/Akt signalling pathway, which has been shown to facilitate CHIKV replication (Thaa et al., 2015). This is particularly noteworthy, as previous studies have demonstrated Sal’s ability to activate PI3K/Akt signalling to promote cell survival and tissue repair in various pathological contexts (Hou et al., 2023). By activating this pathway, Sal may inadvertently create a cellular environment conducive to CHIKV replication. However, the effect of Sal on the PI3K/Akt pathway appears to be context-dependent. For instance, in CoCl_2_-induced HT22 cells, Sal enhances mitochondrial function via activation of the PI3K-Akt-MAPK pathway (Hou et al., 2023). Conversely, in human gastric cancer AGS cells, Sal promotes apoptosis and protective autophagy through the PI3K/Akt/mTOR axis (Rong et al., 2020). Therefore, its precise impact on PI3K/Akt signalling in CHIKV-infected 293T cells requires further experimental validation. Second, our study confirms Sal’s anti-apoptotic effects. RT-qPCR analysis demonstrated that Sal significantly downregulated the expression of key apoptotic markers, including TNF-α, PARP1 and BAX. Molecular docking provided structural support for this activity, revealing a strong binding affinity between Sal and NFKB1 (Vina score: -6.0 kcal/mol). In addition, although CHIKV infection has not been widely associated with ferroptosis, our integrated analysis identified 109 CHIKV-AIN-related targets linked to ferroptosis pathways. Experimental validation showed that Sal downregulated several ferroptosis-associated genes in CHIKV-infected 293T cells, including IL-6, HMOX1 and SIRT1. Docking analysis also confirmed Sal’s interaction with the ferroptosis regulator GPX4 (Vina score: -6.3 kcal/mol). By simultaneously inhibiting apoptosis and ferroptosis, Sal may inadvertently promote the survival of infected cells, potentially allowing CHIKV more time to replicate. This interpretation aligns with previous reports suggesting that pharmacological inhibition of apoptosis can enhance CHIKV production by prolonging host cell viability (Nghia et al., 2020). In this context, the extended survival of infected cells could compensate for the reduced replication efficiency resulting from HSP90AA1 downregulation, thereby sustaining overall viral output.

In conclusion, this study suggests that Sal holds promise as a potential therapeutic agent for CHIKV-induced AIN. However, as these findings are based solely on *in vitro* experiments, further *in vivo* investigations are necessary to validate its efficacy and elucidate its mechanisms of action in a physiological context.

## Data Availability

The datasets presented in this study can be found in online repositories. The names of the repository/repositories and accession number(s) can be found in the article/**Supplementary Material**.

## Supplementary material

Supplementary Table 1 Primer pairs used for target validation.
