# Supplementary material for: Assessing the potential impact of salidroside on Chikungunya virus-induced acute interstitial nephritis via network pharmacology, molecular docking and in vitro experiments
Source: Front Cell Infect Microbiol. 2025 Jul 22;15:1623860. doi: 10.3389/fcimb.2025.1623860 (PMC12321764; doi:10.3389/fcimb.2025.1623860)
Supplement: Supplementary Table 1 — Primer pairs used for target validation. [file Table1.docx]

CHIKV Nsp2 (5'->3')

GGCAGTGGTCCCAGATAATTCAAG

GTACATACCCCACCTAGATCTGTCG

β-Actin (5'->3')

ATCACCATTGGCAATGAGCG

TTGAAGGTAGTTTCGTGGAT

IL6 (5'->3')

TGGGGCTCTTCAAAAGCTCC

AGGAACTATCACCGGATCTTCAA

IL1B (5'->3')

GAAATGCCACCTTTTGACAGTG

GAAATGCCACCTTTTGACAGTG

TNFa (5'->3')

CCAAATGGCCTCCCTCTCAT

GGTGGTTTGCTACGACGTGG

SIRT1 (5'->3')

TGATTGGCACCGATCCTCG

CCACAGCGTCATATCATCCAG

HMOX1 (5'->3')

AGGTACACATCCAAGCCGAGA

CATCACCAGCTTAAAGCCTTCT

PARP1 (5'->3')

GACACCCCGTGCAGACTTG

CCACCACACGTACTGCTCG

BCL2 (5'->3')

ACGTGGACCTCATGGAGTG

TGTGTATAGCAATCCCAGGCA

BAX (5'->3')

AGACAGGGGCCTTTTTGCTAC

AATTCGCCGGAGACACTCG

HSP90AA1 (5'->3')

GACGCTCTGGATAAAATCCGTT

TGGGAATGAGATTGATGTGCAG
